# A Qualitative Study on Formal and Informal Carers' Perceptions of Dementia Care Provision and Management in Malaysia

**DOI:** 10.3389/fpubh.2021.637484

**Published:** 2021-07-21

**Authors:** Michaela Goodson, Emma McLellan, Roshaslina Rosli, Maw Pin Tan, Shahrul Kamaruzzaman, Louise Robinson, Susan Moloney

**Affiliations:** ^1^The Medical School, Newcastle University Medicine Malaysia, Iskandar Puteri, Malaysia; ^2^Population Health Sciences Institute, Faculty of Medical Sciences, Newcastle University, Newcastle upon Tyne, United Kingdom; ^3^Division of Geriatric Medicine, Faculty of Medicine, University of Malaya, Kuala Lumpur, Malaysia

**Keywords:** dementia, Alzheimer's disease, memory loss, care provision, care management, professional awareness, low and middle income country

## Abstract

**Background:** The number of people living with dementia worldwide is increasing, particularly in low- and middle-income countries (LMICs) where little is known about existing post-diagnostic care and support. This study aimed to better understand healthcare provision for people living with dementia in Malaysia, and to identify priorities for providing timely, quality, and accessible care and support to all.

**Methods:** This is a qualitative interview study on care providers and facilitators (health and community care professionals, paid carers, traditional medicine practitioners, faith healers, community leaders, non-governmental organisations). A topic guide, piloted in Malaysia and peer reviewed by all LMIC partners, elicited the understanding of dementia and dementia care and barriers and facilitators to care for people living with dementia and carers, and perceptions of key priorities for developing efficient, feasible, and sustainable dementia care pathways. Verbatim transcription of audio-recorded interviews was followed by iterative, thematic data analysis.

**Results:** Twenty interviews were conducted (11 healthcare professionals, 4 traditional medicine practitioners, and 5 social support providers). The findings indicate that dementia care and support services exist in Malaysia, but that they are not fully utilised because of variations in infrastructure and facilities across the country. Despite a locally recognised pathway of care being available in an urban area, people with dementia still present to the healthcare system with advanced disease. The interviewees linked this to a public perception that symptoms of dementia, in particular, are normal sequelae of ageing. Earlier detection of dementia is commonly opportunistic when patients present to GPs, government clinic staff, and general physicians with other ailments. Dementia may only be identified by practitioners who have some specialist interest or expertise in it. Workforce factors that hindered early identification and management of dementia included lack of specialists, overburdened clinics, and limited knowledge of dementia and training in guideline use. Post-diagnostic social care was reported to be largely the domain of families, but additional community-based support was reported to be available in some areas. Raising awareness for both the public and medical professionals, prevention, and more support from the government are seen as key priorities to improve dementia management.

**Conclusions:** This qualitative study provides novel insight into the availability, delivery, and use of post-diagnostic care and support in Malaysia from the perspective of care providers. The respondents in this study perceived that while there was a provision for dementia care in the hospital and community settings, the different care sectors are largely unaware of the services each provides. Future work should explore how care provision across different service sectors and providers can be supported to better facilitate patient access and referral between primary, secondary, and social care. The importance of supporting families to understand dementia and its progression, and strategies to help them care for relatives was emphasised. There is also a need for broad workforce training and development, at both the postgraduate and undergraduate levels, as well as improved general awareness in the community to encourage earlier help-seeking for symptoms of dementia. This will enable the use of preventive strategies and access to specialist services to optimise care and quality of life for people living with dementia in Malaysia.

## Introduction

Dementia can be defined as a progressive syndrome characterised in a conscious patient by deterioration in memory, thinking, calculation, orientation, comprehension, learning, language, behaviour, judgement, motor tasks, and emotions that has an impact on the ability of an individual to perform everyday activities (1). Around 50 million people worldwide suffer from dementia, with Alzheimer's disease being responsible for 60–70% of the cases and with the remainder comprising vascular dementia, dementia with Lewy bodies, and fronto-temporal dementia diseases (2). The impact of dementia affects not only people who directly suffer from it but also carers, families, and society in general (2). Global estimates suggest 82 million people will be living with dementia in 2030 and 152 million in 2050 with much of this increase in prevalence attributable to ageing populations in low- and middle-income countries (LMICs) (3, 4). Goals for dementia care include early diagnosis to promote optimal management, physical health, cognition, activity, and well-being; detection of accompanying illness; management of behaviour and psychological issues; and provision of long-term support and resource for carers (5).

Malaysia is a multicultural society and a federation of states located in Southeast Asia with a population of over 32.7 million, comprising 7% aged 65 and comprises a federation of states (6). Current estimates suggest that 8.5% of Malaysians aged over 60 years have dementia, yielding an estimated dementia population of 260,345 (7). Studies have shown, however, particularly in Asian countries, that dementia presentation is late because of stigma and acceptance of early symptoms by family members and patients alike as natural sequelae of normal ageing (8).

The Malaysian health care system consists of the public sector, tax-funded and government-run universal services, and a private sector that is funded through private health insurance and out-of-pocket payments from consumers (9). Comprehensive healthcare services range from preventive and primary healthcare to tertiary hospital care (10). In addition, traditional medicines from Chinese and Malay practitioners and products are utilised by large sections of the population. There are around 1,061 Ministry of Health (MOH) and 7,146 private primary care health clinics operating in Malaysia (11). Larger MOH facilities are run by family medicine specialists supported by medical officers, whereas community clinics are staffed by nurses or medical assistants who may have no specialist dementia training. In more remote areas, MOH mobile clinics deliver care (9). Private primary care clinics are largely operated by single-handed or small partnership generalist or specialist doctors, and without the complement of allied health care personnel. Estimates suggest that the public sector provides most (82%) of inpatient care in Malaysia, while the private sector provides most (62%) of the ambulatory care (12–14). While primary care may act as a gate keeper for patients in the MOH system to specialist services, there is no requirement to take this route for private referrals, and patients can self-refer to specialist services or hospitals.

Policies in health and social care for older people in Malaysia currently comprise the National Policy for Older Persons 2011, the National Health Policy for Older Persons 2008, and The Eleventh Malaysia Plan 2016–2020 (15), all of which emphasise active ageing, enablement, and empowerment. These policies do not address any specific age-related conditions, and no individual action plan for dementia currently exists. While healthcare is largely taxation-funded and free for older adults within public systems, social care funding remains highly limited. Institutional care is primarily private- or charitable-sector funded, with limited government-funded places in existence (16). A recent study reports there to be approximately 12 public, 454 non-government organizations (NGO) and 1,019 private nursing homes in Malaysia (17). NGOs also provide some day care services for particular groups, but day to day care for older people /people living with dementia (pwd) is primarily provided by female family members at home, with or without assistance from maids or paid caregivers (18, 19).

Little is known about where services specific to dementia sit within the wider Malaysian health and social care systems or how these services are delivered and accessed. The aim of this study was to illuminate this gap in understanding by exploring current dementia care provisions in Malaysia from the perspective of key stakeholders who either provide or facilitate access to public or private dementia care and support services.

## Materials and Methods

### Study Design

This explorative qualitative study is part of a broader program of research aiming to improve diagnosis and post-diagnostic care for pwd in LMICs (UK National Institute for Health Research Global Health Dementia Prevention and Enhanced Care [DePEC]. A qualitative approach was used, as this enables a richer understanding of views and experiences of the participants on the topic of interest (20). We used the Standards for Reporting Qualitative Research (SRQR) guidelines in writing this manuscript (21) (Supplementary Material). To ensure relevance and cultural acceptability, a purposively sampled panel of eight people with personal or professional experience in dementia care in Malaysia was invited to scrutinise the study design, topic guide, and recruitment strategy (22). The proposed approach to data collection (one-to-one interviews and/or focus groups), such as the study topic guide, was also piloted on a convenience sample of 10 hospital specialists and primary care physicians based in Johor, Malaysia, each with experience in providing dementia services. Following a pilot study using focus groups vs. one-to-one interviews, the interview approach was found to yield richer individual responses with respondents giving more detailed explanations and answers to questions. In focus groups, interruptions from other members of the group made it sometimes difficult for respondents to speak freely and explain their answers to questions from personal experiences. Following minor amendments to language and question format, a final semi-structured topic guide explored three key areas: a) perspectives and practice of participants on dementia care within the wider healthcare system for older adults within Malaysia, b) factors believed to influence the care received by pwd, and c) key priorities for improving the care of pwd in Malaysia (Table 1; Interview topic guide).

**Table 1:** Interview topic guide.

**Section 1: About you**	
1.1 Before we start, can I just ask a few questions about yourself?	Participant background: professional role and role in providing or facilitating dementia care or services
1.2 How would you describe “dementia”?	Participant understanding and description of dementia; where knowledge of dementia was gained
**Section 2: About thehelp seeking behaviours of families/older people who are experiencing symptoms of dementia or who have a diagnosis of dementia**.
2.1 What do patients/families do first? Why?	Health-related help seeking behaviours in general and to older persons' health and symptoms of dementia
2.2 What advice or intervention are people given?	Understanding of explanations offered to older people and families of forgetfulness or behavioural changes.
*2.3When do patients/families seek medical advice or care and what prompts them to do this?*	Understanding of timescale to seeking medical help and what delays presentation (e.g. cultural factors)
2.4 Are there any other factors or reasons why patients present late/ with advanced dementia?	Understanding of other factors (location (rural/urban), convenience, knowledge of services, family finances)
2.5 How can we get people who are experiencing symptoms of dementia into the healthcare system sooner?	What would make a difference; who would be the key stakeholders who could influence change in patient and family behaviours
Section 3: About dementia care in [country]
3.1 Can you tell me what healthcare provision is available in [country] for older people who are experiencing symptoms of dementia or who have a diagnosis of dementia?	Knowledge of a dementia care pathway and availability of dementia facilities (e.g. memory clinics); experience and understanding of how services are accessed.
3.2 Once diagnosed, what is available in terms of treatment?	Knowledge and understanding of pharmacological and non-pharmacological treatment and interventions.
3.3 What is available in terms of social support for people living with dementia and their families?	Understanding of who looks after people with dementia in the community; awareness of social support, training, information resources for families
**Section 4: Factors which influence the care received by people diagnosed with dementia**	
4.1 What things do you think affect the care received by people living with dementia?	Perceived barriers and facilitators related to the pwd; carers; healthcare system; healthcare professionals
4.2 How might care be improved—what needs to change/be put in place	Perceived gaps in current dementia care; aspired ‘ideal' scenarios
4.3 What are the 3 top priority areas to improve the quality of dementia care in your country?	What resources are needed to make change happen; How might these changes being implemented
**Section 5: Workforce capacity, support, training and development**	
5.1 What, if any, dementia specific guidelines are available in your country?	Perceptions/experience of guideline use in practice; relevant to practice; accessibility of guidance
5.2 What training is available to health and social care professionals about older people/ dementia?	Where formal training happens (e.g., core curricula for medical students, nurses, other professionals)
5.3 What about training and information about dementia for families/informal carers?	Understanding of accessibility and uptake
**Section 6: Closing reflections, questions, and close of interview**	
***6.1 Anything else that you'd like to tell or ask me?***	Participant opportunity to add thoughts/ask questions

### Study Population and Sampling Procedure

Eligible participants were (a) providers or facilitators of services offering care and support to pwd or older adults in general, and (b) policy makers and commissioners involved in decision-making regarding healthcare policies and services for older people. We sampled from private, public, and complementary care providers and facilitators based in Kuala Lumpur and Selangor, Malaysia, and included paid or un-paid health and community care professionals, traditional medicine practitioners, faith healers, community leaders, and NGO leads. A purposive sampling frame (22) was developed in collaboration with Malaysia research colleagues to capture a broad range of perspectives from key providers of care to older patients in the Selangor district, and in particular those who were most likely to be involved in diagnosing dementia and delivering dementia care. The sampling frame differentiated the participants by role (e.g., doctor, nurse, allied health professional, community leader), clinical specialty, or area of expertise (e.g., neurology, geriatrics, generalist), sector (e.g., private, public, not-for-profit), organisation type (e.g., social care, primary care, secondary care), and location (e.g., rural, urban). No financial incentive to participate was offered. Given the range of care providers sampled, not all the participants had the same level or kind of knowledge or experience on all issues explored by the topic guide. Therefore, the sampling in this study was purposive and iterative, using “snowballing” to identify additional participants where data was felt to be incomplete (23). Potential participants were approached by email or telephone, provided with a summary of the research, and given the opportunity to ask further questions. All selected participants who were contacted agreed to participate.

### Data Collection

All interviews were conducted by RR, an experienced researcher based in Malaysia, between April 23, 2019 and October 16, 2019. Specialist training and on-going mentorship in qualitative interviewing, qualitative data management, and research governance were provided to RR by EM and SM. Informed consent was taken before an interview began, which included an agreement for the interview to be audio-recorded. The participants were assured of confidentiality. The interviewer (RR) presented herself as a researcher, and the interviewees were informed that their responses would be fully anonymised and that they could withdraw from the interview at any time. The interviews lasted 90 min on average and took place in a private room in the workplace of the participant and during or outside normal working hours. Field notes to aid analysis were made by RR both during and at the end of each interview. The interviews were conducted in English or Bahasa Malaysia, then later transcribed verbatim and fully anonymised. Bahasa Malaysia transcripts were then translated into English, with back translations undertaken on 20% transcripts to ensure accuracy in translation (24). The interviews continued until data saturation was reached, that is, when no new themes were emerging (20, 25).

### Data Analysis

Fully anonymised interview transcripts were analysed iteratively by EM and SM using a thematic analysis approach (25), supported by the use of NVIVO (26). Emergent themes were discussed in depth with RR to ensure the accuracy of interpretation and to guide avenues of further exploration in subsequent interviews. Discussions led to the development of a coding frame, which was then applied independently to a common subset of transcripts by EM, SM, and RR to further check the accuracy of coding and data interpretation and capture of cultural nuances. Differences were reconciled through group discussion, and the coding frame was refined where necessary. EM then coded all the remaining transcripts, adding or revising codes where new themes emerged (20, 25). Emergent findings and the resonance of key themes were continuously checked during group analysis sessions with the wider study team (EM, SM, RR, MG, MP, and LR) to ensure the trustworthiness of interpretation. Final themes and subthemes were organised in relation to the three key areas of interest of the study.

### Ethical Considerations

Prior to data collection, the study was approved by the University of Malaya Medical Centre Medical Ethics Committee (MECID: 201922-7093) in May 2019.

## Results

### Participants

Twenty interviews were undertaken with 11 healthcare professionals (geriatricians, psychiatrists, generalists, general practitioners, nurses, and allied healthcare professionals, e.g., occupational therapists and physiotherapists); five providers of social support (NGO leads, senior citizen association leads, community leaders); and four traditional practitioners (Ayurveda, acupuncture, faith healing) All the interviewees were from the same state, Selangor; and 10 of the clinicians were from the same urban-based, semi-government hospital, while one was from an urban private clinic. Most of the interviewees were based in urban areas, with only two classed as being suburban-based. Fourteen interviews were conducted in English and six in Bahasa Malay (Table 2; Purposive sampling frame and participant demographics).

**Table 2:** Purposive sampling frame and participant demographics.

**Participant Role**	**Sex**	**Ethnicity**	**Years** **qualified**	**Dementia** **training**	**Organisation type and status**	**Location**	**Interview** **language**
Geriatrician 1	F	Chinese	11	Y	Hospital Semi-government	Urban	English
Geriatrician 2	F	Malay	7	Y	Hospital Semi-government	Urban	English
Psychiatrist	M	Indian	6	Y	Hospital Semi-government	Urban	English
General physician 1	M	Chinese	8	N	Hospital Semi-government	Urban	English
General physician 2	F	Chinese	2	N	Hospital Semi-government	Urban	English
Medical officer 1	F	Chinese	10	N	Hospital Semi-government	Urban	English
General Practitioner 1	M	Indian	28	N	Clinic Private	Urban	English
Nurse 1	F	Malay	10	Y	Hospital Semi-government	Urban	Bahasa
Nurse 2	F	Malay	13	Y	Hospital Semi-government	Urban	Bahasa
Occupational Therapist 1	F	Malay	13	N	Hospital Semi-government	Urban	Bahasa
Physiotherapist	M	Malay	12	N	Hospital Semi-government	Urban	English
NGO 1	M	Chinese	N/A	N	NGO Not-for-profit	Urban	English
Senior citizen association 1	M	Chinese	N/A	N	Senior citizen association Not-for-profit	Urban	English
Senior citizen association 2	M	Chinese	N/A	N	Senior citizen association Not-for-profit	Urban	English
Community leader 1	F	Malay	N/A	N	Residential organisation Not-for-profit	Suburban	Bahasa
Community leader 2	F	Chinese	N/A	N	Senior citizen association Not-for-profit	Urban	English
Traditional practitioner 1	M	Indian	31	N	Clinic Private	Urban	English
Traditional practitioner 2	M	Chinese	48	N	Clinic Private	Urban	English
Traditional practitioner 3	M	Malay	34	N	Medical centre Private	Urban	Malay
Traditional practitioner 4	F	Malay	N/A	N	Freelancer Private	Suburban	Malay

### Interview Findings

Findings are presented under three descriptively summarised major themes, each followed by related sub-themes and supporting verbatim quotes. Major themes are knowledge and understanding of available services and management of dementia; factors believed to influence dementia care provision; and priorities for the future–towards improving dementia recognition and timely care.

#### Knowledge and Understanding of Available Services and Management of Dementia

Accounts of the participants revealed a good general understanding of the existing national healthcare system and available healthcare infrastructure in Malaysia. In terms of dementia care, confident description of care provision and pathways into care was limited to the regional level, and detailed description of care processes to immediate personal or organisational context of the participants (e.g., their primary role, clinical discipline, working environment). Outside of this personal context, the participants, particularly those from health vs. social or community settings, tended to demonstrate less clarity of practices and services of each other in relation to dementia care.

##### There Are Multiple Routes Into Specialist Care Services

When exploring how people with symptoms of dementia access dementia care, we found that there are multiple possible routes into specialist medical services, some formal (e.g., in the public sector a referral is required from primary to specialist care), many informal (e.g., collaborative working arrangements between hospital specialists). It was generally accepted that the first point of contact with medical services for people with any kind of ailment, namely, symptoms of dementia, would be in the community-based primary care setting, particularly government health clinics (Klinik Kesihatan, KK) and private general practice.

*I think a lot of times [the first point of contact] will be in the Klinik Kesihatan, family medicine. I think in general practice … because they don't like to wait … they would actually rather just pay a little bit more to see [their own local doctor] so I think … general practitioners, the private GP clinic and also Klinik Kesihatan people* (General Physician 1)

*Probably the first people will be the doctors, the private practitioners … And then from that they go to the specialist, but in this country I think most of them will go to the public the general …, government hospital. The Klinik Kesihatan, now that is where … they seek treatment … that to me is the first line of contact* (NGO 1)

**Figure 1:**
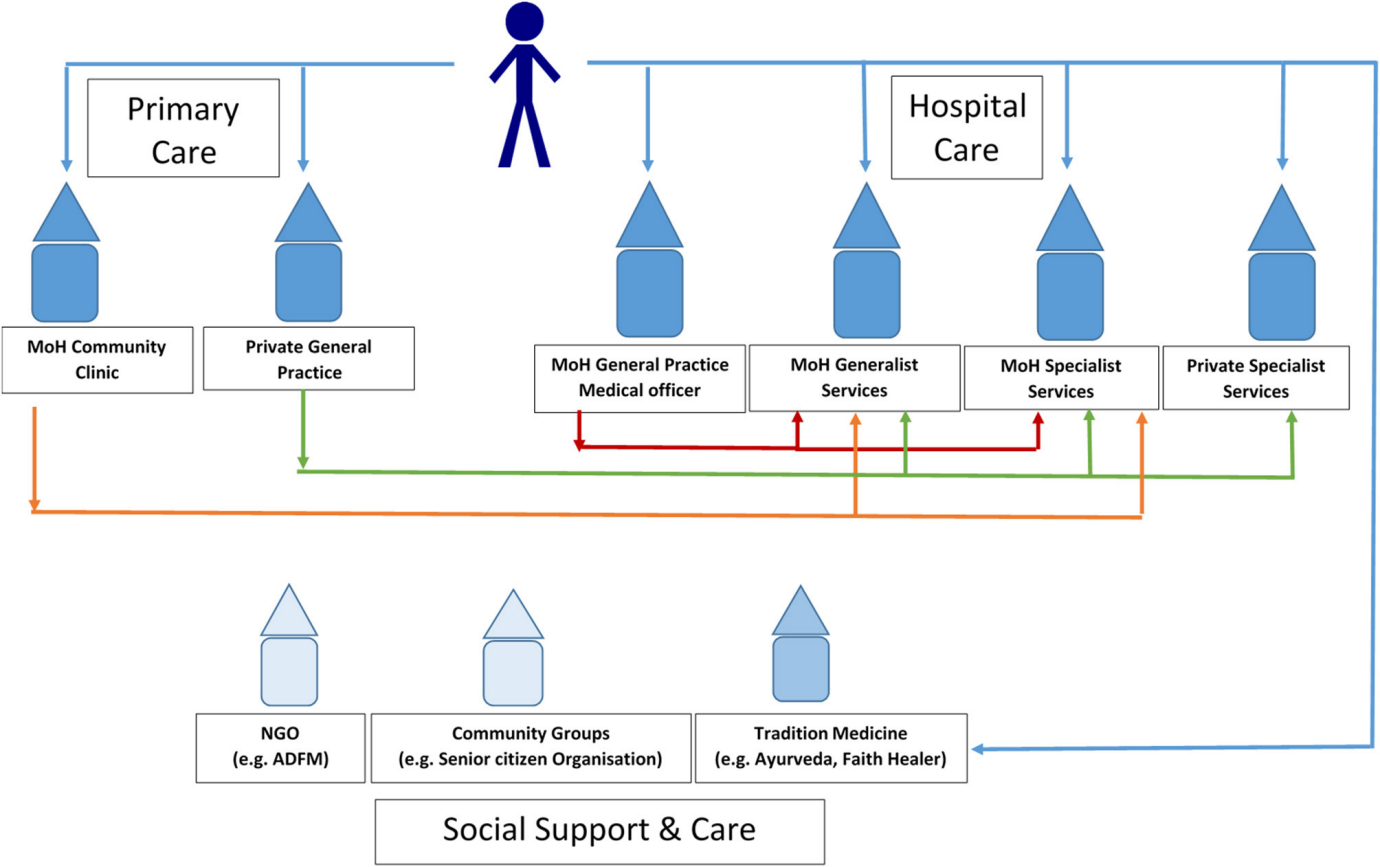
Multiple patient routes to specialist care in Malaysia. The orange, green, and red arrows indicate several referral routes that can take a patient to specialist care. The blue arrows show the multiple routes that can take patients to specialist care and other support services.

However, patients can present to any one of a number of services (Figure 1; Multiple patient routes into specialist care in Malaysia). For example, in addition to community-based primary care services, teaching hospitals also have their own primary care clinics where patients can “walk-in.” These facilities are staffed by family medicine specialists, medical officers (MO), and trainees; and patients requiring further review are referred to a hospital-based specialist. Within teaching hospitals, referral to and between specialities can be more straightforward. Referral from primary care was reported to be more commonplace for KK and rare for private general practice. The participants further confirmed that while a formal primary care referral is typically required to see a specialist doctor in a Ministry of Health hospital, referrals to private specialists are not required. Patients can, therefore, bypass primary care and self-present to private hospital-based specialists.

*Usually when they come to the walk-in they will see the MOs first. And … if the MO finds it difficult to manage they can refer to the specialist for continuation of care. Or they can co-manage the case* (Psychiatrist 1)

*So, we can call any one of them [our geriatricians]… and explain the situation to them ‘look I'm suspecting you know [this] patient … may be having some memory impairment … I would really like your further advice and assessment.' … they will be happy to say, “yes, do write us a referral letter and ask the patient to come to our clinic”* (General Physician 2)

*So the pathway of referral in our clinic, either from another specialist or from a general practitioner. that's how it works, we need a doctor's referral letter. In the private sector, the family members can just bring the patient without referral letter* (Geriatrician 1)

##### Detection of Dementia Is Opportunistic and Specialist Referral Options Inconsistent

The general sense was that people with early signs of memory problems only occasionally present to a clinic for that reason. There is no specific screening for dementia in any out-patient setting, and dementia is more often picked up by chance during consultations with generalist doctors consulting with or assessing patients for something else. Clues to indicate possible dementia may present to a doctor during history taking, for example, or the patient or a relative may directly raise their own concerns.

*It's not like specifically seeing me because of dementia. It's more like seeing me for something else* (General Physician 2)

*Most of the time they don't present as dementia, they present as something else* (General Practitioner)

When symptoms of dementia are detected, the consensus view was that referral to specialists was the best option for assessment and care. However, inconsistency in the availability of services (e.g., memory clinics) and relevant clinical specialists (e.g., geriatrician, neurologist, psychiatrist) was reported. In Malaysia, specialists who have had specific dementia training will predominantly be geriatricians, but sometimes the dementia specialist may be a psychiatrist with geriatrics training. Though not exclusively, geriatricians tend to be attached to a memory clinic, but memory clinics are only available in some areas. In locations without a geriatrician, a referral may be to a psychiatrist or a neurologist where these hospital-based specialists are available. Together, these inconsistencies limit and confuse referral options, making it difficult for primary clinicians to know where or whom to refer a patient to.

*But, in Malaysia there are only a few centers with specialists in dementia. They could be geriatricians, psychiatrists or neurologists. … the person would be very lucky to be able to see a specialist* (Geriatrician 1)

*But they will meet the patient and then after that they don't know who to send the patient [to]. They may have picked up the problem but they don't know how, who or where to send the patient to* (General Physician 1)

*And for a long time we … were referring them to a psychiatrist. But now that we are more aware of the existence of geriatric care within [Hospital 1] we refer to the geriatric department* (General Practitioner 1)

Following differential diagnosis in the hospital setting, assessment for dementia is made using mini-mental state exam (MMSE) (27) and Montreal Cognitive Assessment (MoCA) (28), and CT scan. Confirmation of a dementia diagnosis is given by a dementia specialist. Differentiation between dementia sub-types is not made, as the diagnosis is costly and treatment is usually the same. There is no universal process or register to record and/or share a diagnosis of dementia between specialities, locations, or sectors.

*When [patients] do have symptoms of dementia usually we will investigate further. The usual stuff would be …their blood parameters - things like that or whether they warn in the CT scan. So after things have been worked up usually we will refer to a geriatrician because we have the service here* (MO 1).

##### Post-diagnostic Treatment Is Drug-Focussed With Few Non-pharmacological Options

Like diagnosis, initiation and subsequent management of dementia using drug treatment is largely the domain of geriatricians and psychiatrists. Interviewees from other specialties and allied healthcare roles were aware of the use of drugs in post-diagnostic management of dementia but were less knowledgeable about the drugs used and how they worked. Two main groups of drugs are used, ACE inhibitors and NMDA receptor antagonists, to help with behaviour. However, the use of drugs is not universal amid considerations of potential benefits and harms to the patient. Drugs were not seen to be always effective in slowing disease progression, and their side effects gave cause for concern to those able to prescribe. Some interviewees expressed indifference about the value of drug therapy, given that they do not offer a cure. The high cost of drug therapies was also felt to (negatively) influence family preference for pharmacological treatment of dementia.

*I do not have very much experience using those drugs, because usually we refer them to the geriatrician and it will be started by the geriatrician* (General Physician 1)

*Because there is no medicine that can cure this … Okay you can delay [it] but even that also is not that very effective* (Community Leader 2)

*And the medications that we offer they may not work for a certain percentage of patients and may cause … side effects. They're also very expensive* (Geriatrician 1)

Despite this, it was felt that treatment still tends to focus on medication, since hospitals often do not have the facilities to support non-pharmacological interventions. Furthermore, allied healthcare professionals (e.g., occupational therapists, physiotherapists) and nurses with dementia or elderly care training (e.g., geriatric nurses) tend to be attached to specific specialties. This further limits access to alternative, non-pharmacological therapies, such as those that focus on improving activities of daily living and safety assessment or cognitive training (e.g., reality reorientation therapy).

*All of the geriatrics nurses in this ward have been trained to take care of patients with dementia. We would try to do some treatment like reality orientation, or therapy for instance* (Nurse 1)

*Occupational therapy may give cognitive training exercises, and also cognitive compensatory strategies … also the occupational therapy can do a home safety assessment. … the physiotherapist can teach the family member some simple exercises, to keep the joint supple, reduce pain level, how to control the pain*. (Geriatrician 1)

*That (non-pharmacological treatment) is difficult you see because this requires this requires a lot of teamwork. We don't have that team here. … [in] this hospital the focus is too much on, medication* (Psychiatrist)

##### Counselling and Education of Family Carers Are as Important as Medication

Counselling and education of family carers on dementia and its progression were broadly considered to be of equal, if not greater, importance to medication management. Families shoulder the responsibility of providing care for pwd, and expectations of what this should or could entail to maintain quality of life for pwd were high. For example, as well as personal care, a supportive family was one that also actively maintained social interaction and tailored activities relative to the interests and personal characteristics of pwd. Nonetheless, it was acknowledged how difficult it can be for families to provide such a care environment without support. Educating caregivers, and in some instances the pwd themselves, about dementia and its progression and equipping them with behavioural strategies and coping skills to enable them to care for themselves as well as pwd, were considered by most interviewees as paramount.

*Of course in dementia I think what is even more important than the pharmacological treatment is the non-pharmacological treatment* (Psychiatrist)

*[Be]cause the role of medication in the dementia is … very minimal compared to the understanding of the disease and, preparing for the progression of the disease* (Geriatrician 2)

Providing counselling and education was also widely considered to be a key continuation of the role of dementia “experts” (specialist doctors and geriatric nurses), and hospitals were considered responsible for providing training programs for caregivers. However, time and clinical workload constrain the provision of anything more than basic *ad hoc* counselling, if any at all, of pwd and family members during clinic appointments. Currently, the availability of structured training for family caregivers is understood to be very limited, and written information is more likely to be available in hospitals with established geriatric/psychiatric services. Resources specific to dementia can be available from private organisations, such as drug companies that sponsor educational material. It was suggested that families, particularly those living in urban settings, could access information on dementia from the Alzheimer's Disease Foundation Malaysia (ADFM) (29) or by doing a Google search.

*We also, would also carry out training and carer education. That … is also very important. Let them know that this is a, progressive disease* (General Physician 1)

*I can start … a small bit of counseling about how to look after the person with dementia, but it actually takes, quite a few sessions before the family members are familiar with how to do so* (Geriatrician 1)

*We do offer education. Usually the geriatric clinic nurses or the geriatric nurses on the ward [have responsibility for this]. And also doctors, geriatricians*. (Medical Officer)

*They [family carers] can learn the do's and don'ts when taking care of the patient. That is why the hospitals need to do a program like this* (Community Leader 1)

*[It would] be much easier if the patient can get her diagnosis from the hospital first. The carers can [then] get advice directly on how to take care of their mother* (Traditional Healer 4)

##### Some Social and Community Support Is There, but It Is Not Visible

Similar to education and counselling, the benefits to caregivers of community and social support were broadly recognised. Particular benefits included respite, but in particular the opportunity for caregiver interaction with other people in similar situations, which was felt to help carers to feel less isolated and more resilient through mutual support and shared learning. Alongside the understanding of dementia, such emotional support, rather than practical or monetary support, was felt by some to be all that is required by caregivers. Community-based interviewees suggested that social support is easily available from NGOs but conceded that there is a general lack of awareness of this support among both professionals and the public.

*We really want the public to have this information, so that they can get the benefits out of it, especially to those who have a parent who is already senile, so that they would know what to do* (Traditional Healer 3)

*NGO organizations are there and they all well equipped to [provide support]. The thing is that, [family carers] have to take them there. And family members are not taking [there] … it is because of the time …but the issue with all this system is that, people do not know that they exist* (Traditional Healer 1)

*So there are organized activities but … not many people kind of understand their role in this health and well-being of senior people. Which, there is a great need* (Community Leader 2)

Many interviewees did indeed demonstrate a lack of clarity around what was actually available in terms of social support for pwd and their caregivers. There was awareness of government support for older people, but this was understood to have a more general elderly care focus rather than focus on dementia. Similarly, more general social support specifically for dementia was understood to be minimal, and it was felt that there would likely be geographical variation reliant on having a specialist interested in dementia care in the area. It was suggested that social workers may be able to offer some support services, and, although considered a rare option, some day care centre and residential care can be available from privately managed facilities at a cost. One specialist suggested that referrals can be made to social work for help around funds and carer support, and to ADFM for further information and social support, but whether or not this is common practice is unclear. Lack of awareness and clarity of healthcare professionals about what community-based support is available extended to them not knowing where to look for information about possible services and who provides them.

*Specifically for dementia support I guess it is, still is very very very minimal. Even … support for older adults are, not much. I could not, I couldn't really pinpoint which one is specifically for dementia patient with dementia support* (Geriatrician 2)

*And there are probably support groups but again we don't know where and who's doing it. Yeah. So it's every patient is left to his independent family* (General Practitioner)

#### Factors Believed to Influence Dementia Care Provision

Generally, dementia is presented as having a low profile, with awareness lacking in both public and professional healthcare arenas. While some organised infrastructure and professional expertise exists, and was seen to be improving in some places, there remain significant gaps in workforce capacity and, in particular, workforce capability. Formal training in dementia is confined to doctors and nurses choosing to specialise in geriatrics. National guidelines for dementia care are not routinely used in practice; rather, doctors rely on their own expertise. Families shoulder the responsibility to provide and maintain quality post-diagnostic care to relatives living with dementia, and in the context of limited social support. Despite the acknowledgment of significant personal and financial costs this presents, societal expectation of family-based care remains strong.

##### Public and Professional Understanding and Awareness of Dementia Are Limited

A lack of public knowledge and awareness of dementia was felt by many interviewees to be a key factor that influences both timely help seeking behaviours and the subsequent care that pwd receive. Late presentation, in particular, was highlighted as an issue and was felt to be the result of people not recognising that there is a problem until symptoms become severe. Amid a public perception that older people become senile or lose their memory as a normal part of ageing, families typically seek help only when behaviour becomes too difficult to manage, or their relative has become aggressive. Late presentation of dementia greatly reduces treatment options and the opportunity for early intervention to slow down disease progression. It was also suggested that treatment and follow-up for dementia may not be continued if the family does not understand dementia is a disease. Generally, the interviewees did not express a strong sense of social stigma or cultural taboo attached to the label of dementia itself, but one specialist felt that shame associated with symptoms of dementia could be a factor contributing to delayed help-seeking.

*Lack of awareness. People are aware of the physical health. But they are not very aware of the … dementia or even mental health, you know?* (Community Leader 2)

*You see, when we talk about dementia and Alzheimer's, how many are actually aware about these?* (Nurse 2)

*Sometimes we can see that the carers are not well exposed about dementia* (Occupational Therapist)

*When the person starts to get agitated, like I said when the BPSD is severe, that is when they [families] would seek help* (Nurse 1)

*They [families] wrongly perceive it as a normal part of aging. However they do seek medical attention when the dementia becomes advanced, or moderate, during moderate dementia the person may start having behavioral problems such as aggression* (Geriatrician 1)

Awareness of dementia among non-specialist healthcare professionals, such as primary care doctors, was also reported to be lacking, reducing the potential for early detection of dementia. It was suggested that this also lengthens the time to diagnosis as preliminary investigations, currently undertaken once a patient reaches a geriatric clinic, could be carried out in advance. Lack of dementia awareness in nurses was felt to cause complications with hospital stays, a poor understanding of how dementia impacts the person, and the care needs of pwd.

*I'm not so familiar with the err dementia patient* (Traditional Healer 2)

*Sometimes we can't be very sure that the person is truly senile. We understand that limitation. We don't know if that [is]actually a different disease, because we didn't do any research about that. That is when we need to ask them to go to the hospital. The hospital staff will look after them. They maybe do some scans, investigate the patient's blood and they will check everything. The doctors will help them* (Traditional Healer 4)

*I think again creating awareness. In public and also awareness among healthcare professionals. It's very important* (Medical Officer)

*But I think on the whole there is, generally I'd say, moderate awareness concerning this illness* (Psychiatrist)

*So awareness is very important even among healthcare professionals. Not just awareness of the condition, they need to know where to send the patient to or how to investigate. So they need some guidance as to what's available to them locally in terms of specialist care. And what preliminary investigations they need to do* (Geriatrician 1)

##### Infrastructure and Workforce Capacity for Dementia Care Remains Insufficient

While the provision of dementia services in Malaysia was seen to be improving, it was felt that there still are not enough geriatricians or geriatric services. Most existing geriatricians work in major hospitals in urban areas, but not all hospitals in major cities will have a geriatrician or related specialists. In areas without a geriatrician, getting an appointment to see a visiting geriatrician can take months if the clinic covers a large geographical area. Given the broad scope of geriatrics, doctors in services without a dedicated memory clinic can struggle with excessive workload and overloaded clinics. As in other specialisations where dementia does not take precedence (e.g., neurology and psychiatry), busy clinics tend to be problem-focussed and time-efficient. A patient with dementia can take time to assess, which can be difficult to justify in a busy clinic. Dementia can, therefore, be missed or ignored.

*If we don't have this memory clinic, doctors cannot make a referral to a proper channel. But geriatric clinic is good too … if the hospital … has geriatric clinic services, they will refer this kind of case to the geriatric clinic first. But you see, the geriatrics medicine already covers too many things – their scope is broad. So if lets say we don't have any specific clinic for memory problems, there will be an excessive load of patients and it will be very difficult for doctors to handle the workload* (OT 1)

*To be honest in the very busy clinic. We tend to unfortunately focus only on the primary [condition]. Unless the patient comes in with that actual complaint of memory problems, those symptoms might actually be brushed away* (General Physician 2)

As well as workforce capacity, workforce capability was consistently highlighted as a key factor influencing dementia care. While geriatricians receive good, comprehensive training in the care of older people, such training for other doctors is limited and is often offered as an optional rotation. Subsequently, generalist clinicians (GPs and general physicians) often lack knowledge around diagnosing and treating dementia, despite being involved in caring for pwd. Many students will now get a geriatric rotation, but only basic level dementia training is included as part of the core curriculum for medical studies. Dementia training is also limited in the basic nursing curriculum where gerontology is combined with mental health. More in-depth dementia learning for nurses only occurs when advancing to post basic study.

*[undergraduate training] is very basic. They probably would just learn what dementia is, what are the basic treatments available … so there are several lectures maybe four or five hours' worth of exposure to the topic of dementia in undergraduate days* (Geriatrician 1)

*It [dementia] was just included briefly and slightly. We don't really get to learn about dementia, in term of its care and treatment. We don't really learn those things* (Nurse 2)

Most of the healthcare professionals interviewed had gained knowledge of dementia through their direct experience of working with pwd and from working alongside senior and more experienced colleagues. Uncertainty around the existence of dementia guidelines was evident, and it was suggested that they are not used often. Clinicians, instead, relied on their own experience on dementia or they referred to colleagues with more expertise. Though existing guidelines were felt to be relevant to practice, they were felt to be outdated, aimed specifically at doctors without mention of other multidisciplinary professionals and focus on pharmacological interventions. A further major barrier for dementia capacity building and development is limited budgets, which restrict manpower and resources for delivering care and introducing services.

*But it's more like looking after the elderly patients [where] I gain my knowledge from* (General Physician 2)

*I think there's a guideline for dementia treatment but not very sure. I don't think its been updated yet … I think, guidelines may be useful as a reference but we don't read it like “oh, we got this guideline”. We see the patients [and] we do by experience like “oh, my boss used to do this, do that for dementia. I also do this, do that”* (General Physician 1)

##### Changing Family Circumstances Challenge Traditional Home-Based Care

There is a strong cross-cultural expectation in Malaysia that families take responsibility for the care and well-being of older relatives. The support provided by the family was considered by all the interviewees to be a major factor affecting the care of pwd. However, societal changes to the traditional family structure means that children often now move away to work and do not remain living in close proximity to their parents. Symptoms of dementia may go undetected until a very late stage because of reduced contact. However, living near to or with older relatives does not guarantee family capacity or willingness to provide care for a relative with dementia. Family finances play a key role, and family members may still have to, or wish to, work. Those with more financial resources were considered able to hire a maid to care for pwd; but for others, the need to upkeep the household income may mean leaving pwd alone in the family home. Taking time off work to take relatives to clinic appointments can be costly and inconvenient. For those living in rural areas, there may be a considerable distance to travel and reliance on public transport (i.e., buses and ferries), which can make journeys difficult. These challenges can be a deterrent to attending follow-up appointments.

*Usually in Asian culture society when you retire, or you [are a] pensioner, your family look after you. Your children and grandchildren stay together. But that sometimes is not happening now* (Senior Citizen Association 2)

*One more thing is that, some patients are staying separately from their families. When this happens, nobody will notice the problem when it first starts* (Occupational Therapist)

*But the children are still obliged to take care of their parents with love* (Traditional Healer 3)

In contrast, affluent families tend to live in urban areas with easy access to transport and closer proximity to services. While services provided by government clinics and hospitals are free or greatly subsidised, it was further suggested that those with less money may be reluctant or unable to pay for medication, equipment, or private care. Despite significant difficulties faced by families looking after a relative with dementia being widely acknowledged, social support for dementia was seen to be limited and mostly provided by NGOs. ADFM, for example, was believed to offer free advice, some day care, and training in dementia care for families, healthcare professionals, and English-speaking maids. Access to such support was felt to be influenced by limited availability of services and poor understanding of the role of NGOs.

*It also depends on the financial status of the person with dementia and financial status of the family. Because looking after dementia is an expensive business* (Geriatrician 1)

*A lot of people are unable to afford health care because they don't have money. Not only [for] medications but also equipment* (General Physician 2)

*As for social support, most of it is private NGOs, so we know of the ADFM. Okay, so ADFM has a lot of activities but of course in the end they are only one group and there's only so many people they can reach out to, and then, dementia is a major problem affecting a lot of old people* (General Physician 1)

#### Priorities for the Future—Towards Improving Dementia Recognition and Timely Care

Four key, but interlinked, priorities were identified: raising awareness and knowledge of dementia among the whole population; workforce capacity and capability development; greater family access to culturally sensitive social support services; and raising the profile of dementia through national investment in care services. Together, these priorities were considered essential for encouraging the earlier presentation and efficient diagnosis of dementia, as well as necessary to supporting improvements in the post-diagnostic care of pwd.

##### Raising Public Awareness and Understanding of Dementia Are Paramount

A major priority area for the future is to improve awareness and knowledge of dementia among the whole population. Currently, raising awareness is offered by NGOs and private enterprises. However, it was felt that the government should take the lead to provide a public service or campaign that delivers a broad programme of education tailored to all age groups and pockets of the community felt most likely to have little awareness of dementia (e.g., rural dwelling citizens). Clear guidance should be provided so that people would know what to do if they have a relative showing possible signs of dementia. To encourage earlier help-seeking, a key message to deliver was that dementia is not a normal part of ageing. Clarity should be provided that although there is no cure, there are treatments to slow it down, and that if help is not sought, problems could progress. Several strategies were suggested, such as comprehensive health promotional and educational events and campaigns delivered at a national, regional, and community level, co-delivered by experts in dementia and community and religious leaders. Media coverage of dementia was further suggested in the form of TV and radio shows, and social media platforms.

*So I think the lack of awareness is quite rampant in Malaysia* (General Practitioner 1)

*I think it is about awareness – we need to increase it tremendously … but we don't really reach the rural areas – that is where we actually have to pay more attention in my opinion* (Occupational Therapist)

*But the thing is, the awareness of dementia is still very new. I guess we need to … try to get a lot of general practitioners to be involved in this. So they are a bit aware on the diagnosis and the treatment available. Or the support, care that we can offer this patient and carers* (Geriatrician 2)

*Tell them [public] this is abnormal … so then it will trigger them to say, “why is this happening it's not normal, let's bring him to a doctor”* (General Physician 2)

##### A Trained Specialist Workforce Is Needed to Improve Dementia Care in Malaysia

A second key priority identified was to have more dedicated people to specialise in the care for older people and pwd, namely, doctors, nurses, medical assistants, and all allied healthcare providers. This would require the government to focus on establishing more psychiatric services, and in particular, more neuropsychologists and clinical psychologists to reduce the pressure on psychiatrists. Dementia care in Malaysia would benefit from having more geriatricians, at least one in each state, but ideally one in every hospital. To expand geriatric services, longer-term measures should aim to encourage more specialists into the area. In the shorter term, existing medical professionals, frontline workers, and possibly community workers, could be trained to recognise and pick up dementia. This would help to encourage more visible pathways and efficient referral to geriatric specialists offering dementia services, as well as increasing expertise in diagnosing and caring for pwd in the community. In particular, training general practitioners and general physicians in dementia was seen to be an important step towards improving dementia care, since they are often the first point of contact for patients. Proposed training included informal sharing of expertise by dementia specialists, as well as the development of a training programme by the Ministry of Health and Ministry of Higher Education that would lead to a credible qualification. The need for training extended to providers of dementia care at a community level, including residential care staff or carers who support pwd in a home setting.

*We need, more people who are committed to treat the elderly and the demented patients* (General Physician 2)

*Train the front-liners. You need to train us because if you train us we can pick up patients* (General Practitioner)

*I think at the hospital level, healthcare professionals should be educated on, diagnosing and treating patients with dementia. And that's pretty crucial* (Medical Officer)

*All personnel who are involve in care of dementia patients must be well trained and knowledgeable* (Traditional Healer 3)

##### Families Require Accessible and Culturally Sensitive Social Support

The ideal scenario for the post diagnostic care of pwd was considered to be a good supportive family, with a good understanding of dementia to be able to care for them. Nonetheless, the significant challenges to families were recognised. Subsequently, a key priority in this respect was the improved provision of community and social support for family caregivers. Valuable support is currently provided by NGOs, but the scale of service they can offer is significantly limited. Establishing more community services was, therefore, considered of utmost importance to help improve post-diagnostic care for pwd. Relevant support services proposed include those that: bring medical care into the home (e.g., home visits by specialists for pwd who have developed cognitive and physical impairment); provide respite for carers and stimulation for pwd (e.g., community day centres with activities for pwd); and enhance the quality of life and care for pwd who live at home (e.g., a government-supported domiciliary care system with trained staff).

*People like us we try we try to help out. But the, number of people we can help, is actually minimal* (NGO)

*The third priority is about the home-based care, for those who can't afford to go to the old folks home. What I mean is the community around that area. (…) the community involvement* (Traditional Healer 4)

*We need to establish community centres. We need to establish community service for the elderly …* (Psychiatrist)

*I don't know but if you ask me right, dementia care is a very community kind of, management based kind of disease. Okay, they might come to the hospital once or twice to [review their] general condition, change in medication, but I think the community support is very important. Like community nurse, community Social Worker, pharmacy outside …* (General Physician 2)

Additional support for families should include education and training in dementia and dementia care, and financial support, in the form of an allowance from the government, to help with costs of medications and other aspects of care. Support services need to be sensitive to cultural and religious differences, since these can influence the understanding of symptoms, and because many people continue to visit traditional and faith healers. It was suggested that acknowledgement of such practices could assist cooperative working between conventional medicine and traditional/faith healers. Though not without challenges, such arrangement was considered by some as a potentially positive step that would be beneficial to patients.

*From what I understand, the first thing is to ensure that these care centers or any specific care centers for dementia to provide an environment that can make them feel close to Allah* (Traditional Healer 3)

*We need to try both ways, modern and traditional medicine* (Nurse 2)

*Okay, I think a lot of times people with like oh, a western medicine doctor and they will [be] like oh, I don't work with them. But at the end of the day we have to admit that a lot of people [patients] still work with all these people [traditional/faith healers] and I think that if you make people the enemy “Oh, you are faith healer, you're my … enemy”, then you cannot co-operate with them … I don't believe in the same things as you, but I think we can always cooperate. At the end. this [is] better for patients (General Physician 1)*

*I think if we collectively work together … Okay, the traditional healer has his other ways of massage, this and that, acupuncture, things like that. I think if you can integrate them it's amazing* (General Practitioner)

##### National Investment in Dementia Care and Dementia Prevention Are Paramount

It was felt that dementia care in Malaysia is in its infancy and that the prevalence of dementia is likely to be vastly underestimated. Like other countries across the world, it was felt that Malaysia, as a nation, should be prepared for an ageing population. The profile of dementia and the impact it can have on society, therefore, need to be raised. The interviewees acknowledged that the government has many things to support but with a limited amount of resources. Nonetheless, it was unanimously proposed that increased central investment in dementia care is paramount to improving the capacity and capability to care of the nation for the older population of the country. Further government intervention proposed included establishing a national policy and the development of a national dementia healthcare plan. Finally, prevention was seen to be key, and several strategies enabled by government funding incentives were proposed. These are largely aimed at keeping older people healthy, active, and socialised.

*To make these people realize how much dementia has impacted on our society. In order to, you know, put pressure on the government to give us more money in terms of dementia care* (General Physician 2)

*I think starting from the government they need to allocate more resources to care [for] old people … I think dementia care in Malaysia is still in its infancy. There's a lot more we can do and I would like to do a lot more especially in this unit of mine* (Psychiatrist)

*We suspect the amount, the number of cases as recorded is definitely, definitely not, not projective of what is happening. This is a tip of the iceberg only* (NGO)

*To also educate them that if you follow a lifestyle which is healthy, you can prevent this from developing, you can reduce it …So if we give proper education, we will, first and foremost, be able to reduce, or prevent dementia in the society* (Traditional Healer 1)

## Discussion

This is the first qualitative study undertaken in Malaysia that has captured the views of a range of primary and secondary care providers on access to dementia services, standardised diagnostics, treatment options, and availability and dependence on community services and family support. It has also attempted to understand awareness of dementia among care providers, the perceived influence of traditional beliefs on the management of dementia patients, and knowledge of workforce infrastructure to support dementia care in Malaysia, such as the utilisation of national guidelines. The study was conducted in Selangor, which is an urban area with the highest dementia care service provision in the whole of Malaysia and may, therefore, not represent views of clinicians in Malaysia as a whole, especially where more fragmented and less specialist service provision exists in other states. Nevertheless, the findings show that while a wide variety of services does exist for dementia management in Selangor, there is much room to improve infrastructure and the coordination of services across and within private and public sectors, particularly between primary and secondary care services, so that more standardised and equally accessible care pathways are available to all.

Dementia is a progressive disease and, as such, care needs alteration over time, requiring continual assessment and individual approaches to clinical problems and solutions. In higher income countries (HICs), task shifting out of the primary and secondary care sector into the community has allowed care to be distributed through a larger number of providers (30). When supplemented by national guidelines and a duty of care to provide a standardised care experience, this approach aims to ensure greater equality in care provision (31). Evaluating the conceptualisation of dementia in LMICs where patients have variable opportunities to access care in formal and informal ways is an essential step in understanding how patients and caregivers engage with available dementia services. A recent systematic review of the understanding of dementia in LMICs has found that based on the results of 19 studies, a successful programme of dementia care will need to be developed using a systematic multidisciplinary approach to find acceptable health and community care responses to dementia (32). As demonstrated in the findings, the lack of formal and institutional care for dementia in LMICs places a huge burden on relatives and carers, as well as clinicians, who are managing dementia cases as non-specialists (sometimes with little dementia-specific training) in addition to comprehensive acute and chronic general medical practice (33). While HICs, such as the UK, may have established care pathways for patients with dementia, the application of a similar programme in Malaysia or any other LMIC may not be cost-effective for larger ageing populations with a higher burden of disease, or be manageable within the existing infrastructure. To promote engagement with programmes, cultural contexts, expectations, and shared decision-making are distinct issues that will need to be addressed in the development and design of country-specific care pathways, which, ideally, should also be designed with input from all stakeholders that include patients, carers, medical practitioners, and health/social care ministries to ensure sustainable utilisation of services (32, 34).

In this study, while the respondents had a good understanding of the general healthcare system in Malaysia and their specific roles in the dementia care pathway, they had little understanding of what services may be available in other sectors, how to coordinate care for dementia and other comorbidities, and how to locate or access services other than those they were already using for patients. Similarly, the multiple routes of referral, such as professional and self-referral, made it difficult to follow up on patients and evaluate longer-term outcomes of care. Consequently, the efficiency and improvements to the quality of life as a result of using the dementia services may be difficult to identify and evaluate. Continued involvement and poor knowledge of referral pathways and the additional general lack of awareness of dementia contribute to the issue of late presentation to medical practitioners. Perceptions of the value and efficacy of both pharmacological and non-pharmacological treatments may be influenced by their limited impact on advanced dementia. Late presentation is typical when behavioural problems become unmanageable at home. This delay in help-seeking is compounded by the cultural expectation that younger family members should care for the older generation without external help. From a public health perspective, the presentation of dementia at an advanced stage also makes it difficult to know the exact burden of disease of dementia in Malaysia, particularly so for early cases and for understanding the impact this will have in the future when formal care requirements escalate with disease progression. Unlike Nikmat et al. (8), the respondents did not perceive a current stigma or taboo associated with dementia, suggesting a potential shift in attitudes towards the condition over the past 10 years. However, we have shown that dementia symptoms can still continue to be seen as natural sequelae of ageing in Malaysia. As well as delays in help-seeking, this gap in understanding may also influence timely access to social and community support and uptake of available training, leading to families or paid helpers undertaking care themselves.

Earlier identification of dementia currently relies on opportunistic screening, often when patients are presenting to practitioners with another comorbidity. Since those comorbidities will also require referral and follow-up, there is a risk that families and practitioners may prioritise perhaps more visible problems, such as cardiovascular or respiratory disease over symptoms of dementia, and that these will go unmanaged for longer particularly if there is no clearly identifiable care pathway (35–37). In addition, dementia subtypes are not commonly diagnosed in Malaysia, which may have implications for developing both a local evidence base for treatment outcomes as well as optimising pharmacological management for individual patients. Given that this management is largely under the remit of specialist referral centres, there are likely to be discrepancies across Malaysia where specialists are fewer in number or do not exist. Similarly, non-pharmacological management options may also be limited, such as cognitive stimulation therapy, cognitive rehabilitation, reminiscence, and life story work (38).

While national guidelines for dementia care exist in Malaysia (second edition published in 2009 by Academy of Medicine of Malaysia, 2009) (39), the respondents felt that they were not followed routinely as the evidence base supporting the guidelines in Malaysia was limited and it depended on local service availability, infrastructure, and funding that were not available everywhere. Since resources are not nationally available, the care pathways are very difficult to follow. The guidelines were also felt to be outdated and do not include sufficient non-pharmacological management options. Non-pharmacological interventions may be more economical with improved quality of life outcomes, but they have not been evaluated in Malaysia. Malaysia has not prioritised dementia care over other medical conditions historically and depends heavily on national census and burden of disease data. Unless screening of dementia at an early stage, or early diagnosis, becomes a priority, it is unlikely that a detailed local evidence base for dementia care outcomes will be achievable; and, as a consequence, care will continue to be fragmented across the country with the bulk of services located in Selangor and other larger cities, which, by no means, is solely a LMIC problem (40).

As a clinical specialty, dementia and geriatrics are not perhaps perceived as a glamourous or popular choice for specialty training in Malaysia, as many medical practitioners select specialties where they can gain some element of private practice as well as working in the government system. For general practitioners, it is often not a priority for continuing professional development, so the knowledge and skills for managing dementia patients may be outdated and elementary. The two-tier government/private healthcare approach to service provision means that patients who can afford to will be able to access private services and a wider range of management and care options, but those who can only access government services will be limited to what is accessible in their area. Consequently, national guidelines need to take account of this to ensure some level of standardisation and duty of care in both these sectors. With an ageing population, in order that a sustainable trained workforce is developed for the future, dementia training needs to be expanded in medical curricula at both undergraduate and postgraduate levels. In addition, training for community leaders, religious leaders, and school children to educate for the future would help to improve awareness and address any stigma that currently exists with this disease, as has happened in UK and some LMICs (41–44).

### Strengths and Limitations of the Study and Further Research

A key strength of this study is its use of qualitative methods to provide novel and detailed understanding of dementia care in Malaysia and, in particular, the identification of important areas to improve services. This study provides an overview of the perceptions of formal and informal care providers in Selangor on dementia care in Malaysia. While the experiences of these care providers may not be representative of care providers in Malaysia as a whole, other hospitals and other urban settings, valuable insight is provided from the perspective of those providing dementia care at the frontline of public, private, and not-for-profit services. Further research should build on the current study to collect data in different states to evaluate care provision more broadly, which could also be compared between urban and rural areas. The culturally diverse population of Malaysia includes indigenous populations who may differ in their understanding of dementia and expectations of treatment. A future study on multiple stakeholders, such as carers, early dementia patients, formal and informal carers, ministers, NGO representatives, alternative medicine specialists, and community leaders, would, therefore, be beneficial, examining dementia care challenges at all levels. An integrated study on the culturally diverse population of Malaysia may provide different perspectives on dementia management and indicate needs and priority areas for these groups in the event of improvements on existing dementia management from a national perspective.

## Conclusions

The respondents in this study perceived that while there was provision for dementia care in the hospital and community settings, more could be done to coordinate these services across and within sectors to better facilitate patient access and referral between primary, secondary, and social care. A need for greater emphasis in both postgraduate and undergraduate training in dementia care was reported, as well as improved general awareness in the community to encourage earlier access to care. This will enable the use of preventive strategies and facilities for maximum benefit to potentially optimise quality of life for pwd and to encourage longer-term independent living.

### Implications for Practice

Dementia is a progressive disease, and this study has shown that people with symptoms of dementia in Malaysia often present late to secondary or tertiary care, typically when behavioural issues are too severe to cope with at home. The findings also highlight the already significant pressure on limited specialist dementia services, and on families providing care at home with little to no social support. This is a familiar circumstance for many countries that prompted the 2016 World Alzheimer Report recommendation of a shift towards Western “task-shifted” models of post-diagnostic care (45). Task-shifting involves shifting tasks from specialist, secondary care services to generalist healthcare settings, such as primary care, and/or sharing task between medical and non-medical staff'(30, 45). Task-shifting care models in the United Kingdom have explored shifting aspects of secondary care into the community, but such setup requires considerable infrastructure and buy-in from health professionals and the general public alike. A recent systematic review on post diagnostic care models for dementia has highlighted four primary care-led models from western countries with and without specialist consulting support and case management (46). The review has found that while nurse-led case management partnership models showed the most potential in terms of clinical and cost effectiveness, they may be more costly and needed greater evaluation before they could be universally recommended (46). Other studies have focussed on task-shifting by including a dementia nurse specialist in GP practices (30), but again further research into the remit of the nurse specialist role, cost effectiveness, and benefits of such model is needed. A recent Alzheimer's society report has illustrated how even in a developed country like the UK dementia care is still fragmented and lacks coordination (47). Models of care, such as task-shifting, would need greater health service integration and independent evaluation in Malaysia where care is still largely provided at home and there is little clinical intervention until end-stage dementia.

## Data Availability Statement

The raw data supporting the conclusions of this article will be made available by the authors, without undue reservation.

## Ethics Statement

The studies involving human participants were reviewed and approved by University of Malaya Medical Centre Medical Ethics Committee (MECID: 201922-7093) in May 2019. The patients/participants provided their written informed consent to participate in this study.

## Author Contributions

LR is the principal investigator on the NIHR Global Health DePEC program. MG, LR, and SM contributed to the study design and development and piloting of the topic guide, and provided oversight and coordination of the study. RR conducted the interviews and arranged their transcription and translation. EM, SM, and RR conducted the data analysis. All the authors contributed to the interpretation of the findings. MT and SK provided support in the identification and access to interview the participants. All the authors have approved the final manuscript.

## Conflict of Interest

The authors declare that the research was conducted in the absence of any commercial or financial relationships that could be construed as a potential conflict of interest.

## Abbreviations

PWD: people living with dementia
WHO: World Health Organization
MOH: Ministry of Health
NIHR: National Institute for Health Research
DePEC: Dementia Prevention and Enhanced Care
NGO: Non Governmental Organisation.
